# Gut dysbiosis is associated with difficult-to-treat rheumatoid arthritis

**DOI:** 10.3389/fmed.2024.1497756

**Published:** 2025-01-16

**Authors:** Patricia Ruiz-Limón, Natalia Mena-Vázquez, Isabel Moreno-Indias, Jose Manuel Lisbona-Montañez, Arkaitz Mucientes, Sara Manrique-Arija, Rocío Redondo-Rodriguez, Laura Cano-García, Francisco J. Tinahones, Antonio Fernández-Nebro

**Affiliations:** ^1^The Biomedical Research Institute of Malaga and Platform in Nanomedicine (IBIMA BIONAND Platform), Málaga, Spain; ^2^Unidad de Gestión Clínica de Endocrinología y Nutrición, Hospital Universitario Virgen de la Victoria, Málaga, Spain; ^3^CIBER Fisiopatología de la Obesidad y Nutrición (CIBEROBN), Instituto de Salud Carlos III, Madrid, Spain; ^4^UGC de Reumatología, Hospital Regional Universitario de Málaga, Málaga, Spain; ^5^Departamento de Medicina. Universidad de Málaga, Málaga, Spain

**Keywords:** rheumatoid arthritis, gut microbiota, disease modifying antirheumatic drugs, *Firmicutes/Bacteroidetes* ratio, inflammation

## Abstract

**Background:**

Difficult-to-treat rheumatoid arthritis (D2T RA) refers to a subset of patients who fail to achieve adequate disease control after the use of two or more biological or targeted synthetic disease-modifying antirheumatic drugs (b/tsDMARDs) with different mechanisms of action, while maintaining active inflammatory disease. This presents a therapeutic challenge and highlights the need to explore contributing factors such as the potential role of the gut microbiota. Therefore, the aim of this study was to analyze the gut microbiota and inflammation in patients with D2T RA in comparison to patients with easy-to-treat RA (E2T RA).

**Objective:**

To analyze the gut microbiota and inflammation in patients with D2T RA.

**Methods:**

We performed an observational study of a prospective cohort between 2007 and 2011 and analyzed the gut microbiota. In 2022, we identified 2 extreme patient phenotypes: (1) D2T RA, which was defined as failure of ≥2 biological or targeted synthetic disease-modifying antirheumatic drugs (b/tsDMARDs) (with different mechanisms of action) plus signs of active disease; and (2) easy-to-treat RA (E2T RA), i.e., stable disease managed with a single treatment. The gut microbiota was analyzed using 16S rRNA gene sequencing; bioinformatics analysis was performed using QIIME2, and its functionality was inferred through PICRUSt. We recorded data on clinical findings, inflammation, and cytokines. A Cox multivariate analysis was performed to identify factors related to D2T RA.

**Results:**

The study population comprised 39 patients: 13 (33%) with D2T RA and 26 (66%) with E2T RA. The families *Lachnospiraceae* and *Pasteurellaceae*, and their genera *Coprococcus* and *Haemophilus* were more abundant in E2T RA patients, while the genus *Megasphaera* was more abundant in D2T RA patients. The *Firmicutes*/*Bacteroidetes* ratio decreased in D2T RA patients. The metabolic profile of the gut microbiota was characterized by differences in Degradation/Utilization/Assimilation pathway and the Biosynthesis pathway. The factors associated with D2T RA were inflammatory activity according to DAS28-ESR (HR, 2.649; *p* = 0.013), prednisone (HR, 3.794; *p* = 0.008), and the *Firmicutes*/*Bacteroidetes* ratio (HR, 0.288; *p* = 0.033).

**Conclusion:**

The composition of the gut microbiota of patients with D2T RA differed from that of E2T RA patients, as did the metabolic pathways.

## Introduction

Rheumatoid arthritis (RA) is an immune-mediated chronic inflammatory disease characterized by involvement of the joints and other tissues. It causes pain, stiffness, and loss of mobility. Although there is no cure for RA, affected patients can be treated with a combination of conventional synthetic disease-modifying antirheumatic drugs (scDMARDs), biological DMARDs (bDMARDs), and targeted synthetic DMARDs (tsDMARDs) (1). Despite this therapeutic arsenal, between 15 and 30% of patients with RA have disease that is difficult to treat (D2T RA) and do not achieve remission or low disease activity (2).

According to the “*European Alliance of Associations for Rheumatology*” (EULAR) (3), patients with RA are considered D2T if at least 2 biological or targeted synthetic disease-modifying antirheumatic drugs (b/tsDMARDs) (with different mechanisms of action) have failed after previous failure of a csDMARD (unless contraindicated). Furthermore, the patient must have signs of active/progressive disease in which management of signs and/or symptoms is problematic for the rheumatologist and/or doctor.

Various studies have attempted to identify factors associated with a poor response to biologics in RA (4–7). Some of these factors include, once poor adherence and inadequate dosing have been excluded, higher autoantibody levels (6), a greater number of comorbid conditions (4, 6, 7), and more marked inflammatory activity at initiation of therapy (5–7). However, other studies have shown that patients with marked inflammatory activity can respond favorably to bDMARDs (6).

While the etiology of RA is not fully clear, it has been suggested that the disease results from the interaction between genetic and environmental factors (8). In recent years, several studies have focused on the gut microbiota as a major pathogenic factor in RA (9). Many studies have suggested that the degree of dysbiosis differs between patients with RA and controls and that microbial diversity is poorer in patients with RA than in controls. Similarly, intestinal dysbiosis and specific uncommon and harmful bacterial lineages have been associated with continuous high inflammatory activity or with the presence of factors affecting the severity of RA (9–12). Data on the effect of DMARDs on the gut microbiota in RA are limited (13), and no studies to date have specifically evaluated the gut microbiota of patients with D2T. In addition, no evidence is available on whether there is an association between continuously high inflammatory activity and high levels of proinflammatory cytokines in patients with D2T RA. Therefore, the primary objective of the present study was to compare the gut microbiota, cumulative inflammatory activity, and other clinical characteristics between patients with D2T RA and patients who respond well to therapy in order to identify microbial profiles and other factors associated with D2T RA.

## Methods

### Design, data source, and scope

The data for this controlled cross-sectional study came from a prospective cohort of incident cases recruited between 2007 and 2011 in the Department of Rheumatology, Hospital Universitario Regional de Málaga, Málaga, Spain (9). All the patients were aged ≥18 years, fulfilled the 2010 criteria of the American College of Rheumatology/European League Against Rheumatism for RA (14), and had been diagnosed and treated for the first time during the 12 months after onset of their disease. All participants provided their written informed consent before inclusion. The study was conducted according to the principles of the Declaration of Helsinki, and the study protocol was approved by the Ethics Committee of Málaga (Project identification code 4/2016, P19).

### Participants and study protocol

Since the creation of the prospective cohort (2007–2011), all patients have been followed up at the outpatient clinic every 3–6 months by a rheumatologist using a systematic clinical data collection protocol. The data collected included inflammatory activity and physical function throughout follow-up.

During 2016–2018, all the patients in the cohort consented to a relevant modification to the protocol aimed at new, broader study objectives that enabled, among other options, the present study. Peripheral venous blood samples were collected after overnight fast, and fecal samples were refrigerated immediately and transported to the laboratory, where they were stored at −80°C for subsequent analysis. The date of this visit was the index date.

At the last visit in 2022 (final visit in the present study), we identified 2 groups of patients with extreme RA phenotypes: (1) a group comprising patients with difficult-to-treat RA (D2T RA); and (2) a group of easy-to-treat RA (E2T RA) patients at a 2:1 ratio, matched by age, sex, and time since diagnosis.

D2T RA was defined according to the EULAR criteria for D2T RA, as follows: failure of ≥2 biological or targeted synthetic disease-modifying antirheumatic drugs (b/tsDMARDs) (with different mechanisms of action) after failure of a csDMARD (unless contraindicated); signs suggestive of active/progressive disease; and management of signs and/or symptoms perceived as problematic by the rheumatologist and/or the patient (3). E2T RA was defined as treatment throughout follow-up with a single csDMARD and neither active nor progressive disease. Following the EULAR recommendations (3), active/progressive disease was defined as ≥1 of the following: at least moderate disease activity (according to validated composite measures including 28-joint Disease Activity Score for Rheumatoid Arthritis with erythrocyte sedimentation rate [DAS28-ESR] >3.2), signs and/or symptoms suggestive of active disease, inability to taper glucocorticoid treatment (below 7.5 mg/day prednisone or equivalent), rapid radiographic progression, and symptoms that diminish quality of life.

### Outcome measures

On the index date, we collected demographic, clinical, and treatment-related data, including comorbid conditions associated with traditional cardiovascular risk factors (smoking, obesity, arterial hypertension, diabetes mellitus, history of cardiovascular disease, and sedentary lifestyle).

Similarly, on the index date, we evaluated inflammatory activity at the visit and calculated the cumulative activity. Activity was estimated as an arithmetic mean of all the values collected regularly since diagnosis (time-averaged disease activity). Inflammatory activity was measured using the DAS28-ESR (range, 0–9.4) (15). A DAS28-ESR value >3.2 was considered high and ≤3.2 was considered low. The erythrocyte sedimentation rate (ESR; mm/h) was measured. High-sensitivity C-reactive protein (hsCRP; mg/L) was measured for all participants using nephelometry (MMAGE-Immunochemistry Systems, Beckman Coulter, Brea, CA, United States). Physical function on the index date was assessed (average value) using the Health Assessment Questionnaire (HAQ).

We recorded the presence of autoantibodies and their titers, as follows: rheumatoid factor (positive if >10 IU/mL) and anti–citrullinated peptide antibody (positive if >20 IU/mL), as well as the presence or absence of radiologic erosions. We recorded all csDMARDs, such as methotrexate, leflunomide, and sulfasalazine, and bDMARDs, such as tumor necrosis factor inhibitors (anti-TNF), tocilizumab, abatacept, and rituximab. We also included tsDMARDs, such as the Janus kinase inhibitors (JAKi) tofacitinib and baricitinib, and glucocorticoids. Adherence to the Mediterranean diet was evaluated using a validated questionnaire in which adherence was defined as a score of >9 out of 14 (16).

### Plasma levels of interleukins, human growth factors, and lipoproteins

Inflammatory mediators such as TNF-α, IL-1β, and IL-6 in plasma were quantified using enzyme-linked immunosorbent assay (ELISA) Quantiglo kits (R&D Systems Inc., Minneapolis, United States) according to the manufacturer’s instructions. Plasma levels of insulin-like growth factor I were analyzed using ELISA (Mediagnost GmbH., Tuebingen, Germany). Malondialdehyde-oxidized low-density lipoprotein (LDL) was also measured in plasma using an ELISA kit (Biomedica GmbH., Vienna, Austria).

### Gut microbiota analysis

DNA was extracted using the QIAamp DNA Stool Mini Kit (Qiagen, Hilden, Germany) according to the manufacturer’s instructions. The concentration and purity of DNA were determined using a Nanodrop spectrophotometer (Nanodrop Technologies, Wilmington, DE, United States). Ribosomal 16S RNA (16S rRNA) gene sequences were amplified from DNA using the Ion 16S Metagenomics Kit (Thermo Fisher Scientific, Waltham, MA, United States). The kit includes 2 primer sets (V2-4-8 and V3–6, 7–9) that selectively amplify the corresponding hypervariable regions of the 16S region in bacteria. Libraries were built with the Ion Plus Fragment Library kit (Thermo Fisher Scientific). Barcodes were added to each sample using the Ion Xpress^™^ Barcode Adapters kit (Thermo Fisher Scientific). Emulsion PCR and sequencing of the amplicon libraries were performed on an Ion 530 chip (Ion 530^™^ Chip Kit) using the Ion Chef System and Ion Torrent S5^™^ system (Ion 510^™^/520^™^/530^™^ Kit-Chef, Thermo Fisher Scientific), respectively, according to the manufacturer’s instructions.

### Bioinformatic analysis

Base calling and run demultiplexing were performed using Torrent Suite™ Server software (Thermo Fisher Scientific), version 5.4.0, with default parameters for targeted sequencing of 16S (bead loading ≤30, key signal ≤30, and usable sequences ≤30). Quality sequences were further translated into amplicon sequence variants (ASVs) using DADA2 with adapted parameters for Ion Torrent data within the open-source Quantitative Insights into Microbial Ecology software (QIIME2, version 2022.2) (17). Taxonomic assignment was performed through clustering with VSEARCH and the reference base Greengenes version 13_8 at 97% identity. Before analysis, samples with fewer than 1,500 sequences, features with a count sum of less than 10 across all samples, those presented only in one sample, mitochondrial features, and features unidentified at the phylum level were removed in the pre-processing step. Alpha and beta diversities were evaluated using the core-metrics-phylogenetic plugin in QIIME2 after rarefaction to the minimum number of sequences per sample. Alpha diversity was assessed through different indexes, including Pielou-evenness, Faith-PD, observed-features, and Shannon index. Statistically significant differences in alpha diversity between groups were determined by using the Kruskal-Wallis test with a significance level of *p* = 0.05. The beta diversity was assessed using non-phylogenetic Bray–Curtis dissimilarity index. The structure of the gut microbial community of the different groups was examined using PCoA plots for Bray-Curtis distances. The significance of variations among groups was determined using permutational multivariate analysis of variance (PERMANOVA) with a *p*-value of 0.05. Linear discriminant analysis (LDA) Effect Size (LEfSe) was used to identify potential biomarker taxa. LEfSe was performed on the online Galaxy web application^1^ (18), where data were normalized using counts per million (CPM). The nonparametric factorial Kruskal–Wallis sum rank test was used to detect differential abundant features (at genera, family, class, order, and phylum level) within the two groups. As a last step in the LEfSe analysis, LDA was used to determine the effect size of each differentially abundant feature. The differences in microbial relative abundance were considered significant when the LDA < 2 and *p* < 0.05 (19). The cladogram from the LEfSe method indicates the phylogenetic distribution representing differentially abundant taxonomic groups. The size of each node represents its relative abundance. The Phylogenetic Investigation of Communities by Reconstruction of Unobserved States plugin (PICRUSt2) (20) was used to predict metagenome function within QIIME2 with the DADA2 output. MetaCyc pathways (21) were normalized within QIIME2 and further analyzed using the open-source software STAMP (Statistical Analysis of Metagenomics Profiles) with Welch’s *t* test option (22).

### Statistical analysis

A descriptive analysis of the main outcome measures was performed. Values are expressed as frequencies and percentages or as mean (standard deviation [SD]) or median (interquartile range [IQR]), as applicable. Normality was assessed using the Kolmogorov–Smirnov test. We compared clinical and laboratory characteristics and inflammatory activity between patients with D2T RA and patients with E2T RA using the Pearson χ^2^ test or the *t* test, as applicable. A multivariate analysis was performed, as was a Cox regression analysis, to identify factors associated with D2T RA adjusted for disease duration. The variables entered into the models were those that proved to be significant in the bivariate analysis and of clinical interest. Statistical significance was set at *p* < 0.05. The statistical analyzes were performed using IBM SPSS Statistics for Windows, Version 28 (IBM Corp., Armonk, NY, United States).

## Results

We included 39 patients with RA, of whom 13 (33%) were D2T RA and 26 (66%) were E2T RA. Most were women (82%), with a mean (SD) age of 55.1 (11.6) years on the index date. In line with the definitions, at the end of follow-up (final visit), all D2T RA patients had been treated with at least 2 different lines of biologic therapy, whereas E2T RA patients had only received treatment with methotrexate, except for 1 patient who was treated with leflunomide owing to intolerance to methotrexate at 10 mg/wk.

### Epidemiological and clinical characteristics and comorbid conditions

Table 1 shows the clinical and epidemiological characteristics of D2T RA patients and E2T RA patients on the index date. Data from both groups were consistent for most of the clinical-epidemiological characteristics and comorbid conditions, except that erosions were more frequent in D2T RA patients than in E2T RA patients (*p* = 0.021). There were no significant differences in the studied comorbidities related to cardiovascular risk, osteoporosis, fibromyalgia, or depression between the two groups. As for treatment, most D2T RA patients were receiving treatment with an anti-TNF agent on the index date (69.2%) or an IL-6 inhibitor (23.1%) (Table 1).

**Table 1:** Clinical and epidemiological data on the index date.

Variable	D2T RA *n* = 13	E2T RA *n* = 26	*p*-value
**Epidemiological characteristics**			
Age, years, mean (SD)	52.9 (12.4)	56.7 (11.0)	0.323
Female sex; *n* (%)	11 (84.6)	21 (80.8)	0.768
White race, *n* (%)	13 (100.0)	26 (100.0)	1.000
Smoking			0.598
Never smoked, *n* (%)	5 (38.5)	13 (50.0)	
Ex-smoker, *n* (%)	5 (38.5)	6 (23.1)	
Active smoker, *n* (%)	3 (23.1)	7 (26.9)	
**Anthropometric data**			
BMI (kg/m^2^), mean (SD)	27.9 (4.4)	26.8 (4.0)	0.466
Abdominal circumference, mean (SD)	91.0 (10.4)	88.0 (12.7)	0.466
Hip circumference, mean (SD)	104.8 (11.1)	102.7 (8.8)	0.533
Waist-hip index, mean (SD)	0.86 (0.06)	0.85 (0.09)	0.652
**Comorbid conditions**			
Arterial hypertension, *n* (%)	4 (30.8)	7 (26.9)	0.801
Diabetes mellitus, *n* (%)	1 (3.8)	0 (0.0)	0.474
Dyslipidemia, *n* (%)	4 (30.8)	5 (19.2)	0.420
Obesity WHO (BMI ≥ 30), *n* (%)	5 (38.5)	6 (23.1)	0.314
Cardiovascular disease, *n* (%)	2 (15.4)	4 (15.4)	1.000
Osteoporosis, *n* (%)	2 (15.4)	3 (11.5)	0.735
Fibromyalgia, *n* (%)	0 (0.0)	2 (7.7)	0.305
Depression, *n* (%)	1 (7.7)	0 (0.0)	0.333
Anxiety-depressive syndrome, *n* (%)	2 (15.4)	2 (7.7)	0.455
**Clinical characteristics**			
Time since diagnosis of RA, months, median (IQR)	101.3 (85.9–142-0)	100.7 (77.5–132.4)	0.618
Diagnostic delay, months, median (IQR)	13.8 (6.3–24.0)	10.3 (4.6–22.6)	0.627
Erosions, *n* (%)	11 (84.6)	12 (46.2)	0.021
RF > 10, *n* (%)	11 (84.6)	23 (88.5)	0.735
ACPA > 20 U/mL, *n* (%)	12 (92.3)	21 (80.8)	0.401
Elevated ACPA > 340 U/mL, *n* (%)	6 (46.2)	7 (26.9)	0.199
**Treatment**			
Synthetic DMARDs, *n* (%)	11 (84.6)	26 (100.0)	0.040
Methotrexate, *n* (%)	6 (46.2)	25 (96.2)	<0.001
Leflunomide, *n* (%)	3 (23.1)	1 (3.8)	0.062
Sulfasalazine, *n* (%)	1 (7.7)	0 (0.0)	0.152
Hydroxychloroquine, *n* (%)	1 (7.7)	0 (0.0)	0.152
Biologic DMARDs, *n* (%)	13 (100.0)	0 (0.0)	<0.001
Anti-TNF, *n* (%)	9 (69.2)	0 (0.0)	<0.001
Anti–IL-6, *n* (%)	3 (23.1)	0 (0.0)	0.011
Tofacitinib, *n* (%)	1 (7.7)	0 (0.0)	0.151
Glucocorticoids, *n* (%)	4 (30.8)	1 (3.8)	0.018
Dose of glucocorticoid, median (IQR)	0.0 (0.0–5.0)	0.0 (0.0–0.0)	0.040

RA, rheumatoid arthritis; SD, standard deviation; BMI, body mass index; WHO, World Health Organization; IQR, interquartile range; RF, rheumatoid factor; ACPA, anti–citrullinated peptide antibody; DMARD, disease-modifying antirheumatic drug.

However, at the final visit (see Table 2), most of the 13 patients with D2T RA were receiving rituximab (30.8%) or tofacitinib (23.1%) after a mean (SD) of 2.6 (1.3) switches of biologics, with a mean (SD) retention period of 95.8 (56.3) months. In patients with D2T RA, the main reason for switching biological or targeted synthetic disease-modifying antirheumatic drugs (b/tsDMARDs) was loss of efficacy (18/34 treatments used [52%]), followed by insufficient response (11/34 [32%]) and nonserious adverse events (5/34 [14%]).

**Table 2:** Treatments at the index date (2016–2018) and the final visit (2022) in D2T RA and E2T RA patients.

Biologic	Time point*	D2T RA *n* = 13	E2T RA *n* = 26	*p*-value
csDMARD, *n* (%)	Index-date	11 (84.6)	26 (100.0)	0.040
	2022	10 (76.9)	26 (100.0)	0.011
Methotrexate, *n* (%)	Index-date	6 (46.2)	25 (96.2)	<0.001
	2022	5 (38.5)	25 (96.2)	<0.001
Leflunomide, *n* (%)	Index-date	3 (23.1)	1 (3.8)	0.062
	2022	3 (23.1)	1 (3.8)	0.062
Sulfasalazine, *n* (%)	Index-date	1 (7.7)	0 (0.0)	0.152
	2022	1 (7.7)	0 (0.0)	0.152
Hydroxychloroquine, *n* (%)	Index-date	1 (7.7)	0 (0.0)	0.152
	2022	1 (7.7)	0 (0.0)	0.152
bDMARD, *n* (%)	Index-date	13 (100.0)	0 (0.0)	<0.001
	2022	13 (100.0)	0 (0.0)	<0.001
Anti-TNF, *n* (%)	Index-date	9 (69.2)	0 (0.0)	<0.001
	2022	2 (15.4)	0 (0.0)	0.040
Anti–IL-6, *n* (%)	Index-date	3 (23.1)	0 (0.0)	0.011
	2022	2 (15.4)	0 (0.0)	0.040
Rituximab, *n* (%)	Index-date	0 (0.0)	0 (0.0)	–
	2022	4 (30.8)	0 (0.0)	0.001
Tofacitinib, *n* (%)	Index-date	1 (7.7)	0 (0.0)	0.151
	2022	3 (23.1)	0 (0.0)	0.011
Baricitinib, *n* (%)	Index-date	0 (0.0)	0 (0.0)	–
	2022	1 (7.7)	0 (0.0)	0.151
Abatacept, *n* (%)	Index-date	0 (0.0)	0 (0.0)	–
	2022	1 (7.7)	0 (0.0)	0.151

RA, rheumatoid arthritis; D2T, difficult-to-treat; E2T, easy-to-treat; DMARD, disease-modifying antirheumatic drug; csDMARD, conventional synthetic DMARD; bDMARD, biologic DMARD. *Treatments recorded at two specific time points: the index date (2016–2018, when blood and stool samples were collected) and the final visit (2022, when the extreme phenotypes were defined).

In contrast, as expected, the 26 E2T RA patients maintained the same csDMARDs throughout follow-up. Similarly, a larger number of patients with D2T RA were receiving glucocorticoids on the index date (*p* = 0.018) and at a higher median dose than the E2T RA patients (*p* = 0.040).

### Study of inflammatory factors and cytokines

Of the 39 patients included, 28 (71.8%) were in remission or with low disease activity at the index date according to their DAS28-ESR values, and 30 (76.9) had maintained an average DAS28-ESR value indicating low activity. D2T RA patients had higher average DAS28-ESR values at the cut-off than the E2T RA patients (Table 3). The same was true of physical functioning according to the HAQ. However, laboratory values were generally similar for both groups, except for some notable differences, such as homocysteine (*p* = 0.010) and CRP (*p* = 0.029), which were superior in D2T RA.

**Table 3:** Inflammatory factors and cytokines in D2T RA and E2T RA.

Variable	D2T RA *n* = 13	E2T RA *n* = 26	*p-*value
**Inflammatory activity**			
DAS28 average value, mean (SD)	3.3 (1.1)	2.4 (0.6)	0.003
Remission-low activity, *n* (%)	6 (46.2)	22 (84.6)	0.012
Moderate-high activity, *n* (%)	7 (53.8)	4 (15.4)	0.012
DAS28 at index-date, mean (SD)	3.0 (0.4)	2.5 (0.5)	0.016
Remission-low activity, *n* (%)	7 (53.8)	23 (88.5)	0.018
Moderate-high activity, *n* (%)	6 (46.2)	3 (11.5)	0.018
HAQ average value, mean (SD)	1.0 (0.7)	0.5 (0.5)	0.026
HAQ at index date, mean (SD)	1.1 (0.3)	0.8 (1.1)	0.516
**Laboratory parameters**			
ESR, mm/h, median (IQR)	16.0 (5.57–23.0)	12.0 (8.7–17.2)	0.603
hsCRP, mg/L, mean (SD)	5.6 (3.4)	3.7 (1.9)	0.029
Hemoglobin, g/dL, median (IQR)	12.8 (11.5–13.7)	13.5 (12.4–14.1)	0.066
Leukocytes, 10^9^/L, median (IQR)	5960.0 (4695.0–7380.0)	6375.0 (5150.0–6897.5)	0.848
Platelets, 10^9^/L, mean (SD)	291769.2 (92585.2)	240500.0 (53234.7)	0.083
Creatinine, mg/dL, mean (SD)	0.7 (0.1)	0.7 (0.1)	0.443
Total cholesterol, (mg/dL), mean (SD)	197.5 (28.8)	199.0 (45.3)	0.914
LDL cholesterol, mg/dL, median (IQR)	113.0 (90.0–135.0)	109.8 (92.9–126.7)	0.988
HDL cholesterol, mg/dL, median (IQR)	60.0 (53.5–75.0)	59.5 (54.0–81.5)	0.980
Triglycerides, mg/dL, median (IQR)	86.0 (66.0–112.0)	72.5 (64.0–89.6)	0.384
Homocysteine, mg/L, median (IQR)	18.1 (13.7–20.9)	12.0 (10.5–13.7)	0.010
Interleukins, lipoproteins, and human growth factors			
IL-6, pg./mL, median (IQR)	13.8 (9.9–60.2)	6.6 (4.1–10.6)	0.031
IL-1β, pg./mL, median (IQR)	4.2 (4.1–4.3)	4.3 (4.1–4.4)	0.473
TNF-α, pg./mL, median (IQR)	8.6 (3.7–267.5)	4.6 (3.6–6.1)	0.085
IGF-1, pg./mL, median (IQR)	138.5 (71.2–316.5)	177.9 (108.5–217.2)	0.872
Oxidized LDL (IU/mL), median (IQR)	1.0 (0.5–3.7)	3.3 (0.8–5.7)	0.343

RA, rheumatoid arthritis; DAS28, 28-joint Disease Activity Score; SD, standard deviation; D2T, difficult-to-treat; E2T, easy-to-treat; HAQ, Health Assessment Questionnaire; HDL, high-density lipoprotein; hs-CRP, high-sensitivity C-reactive protein; IGF-1, insulin-like growth factor-1; ESR, erythrocyte sedimentation rate; IL-6, interleukin 6; LDL, low-density lipoprotein; TNF, tumor necrosis factor.

Regarding proinflammatory cytokines, D2T RA patients had higher levels of IL-6 (*p* = 0.031), and numerically higher levels of TNF-*α* (*p* = 0.085). In the case of lipoproteins and human growth factors, the values remained similar in both groups. Additionally, D2T RA patients were similar to those with E2T in their adherence to the Mediterranean diet (69.2% vs. 73.0%; *p* = 0.901).

### Comparison of gut microbiota between groups

**Figure 1:**
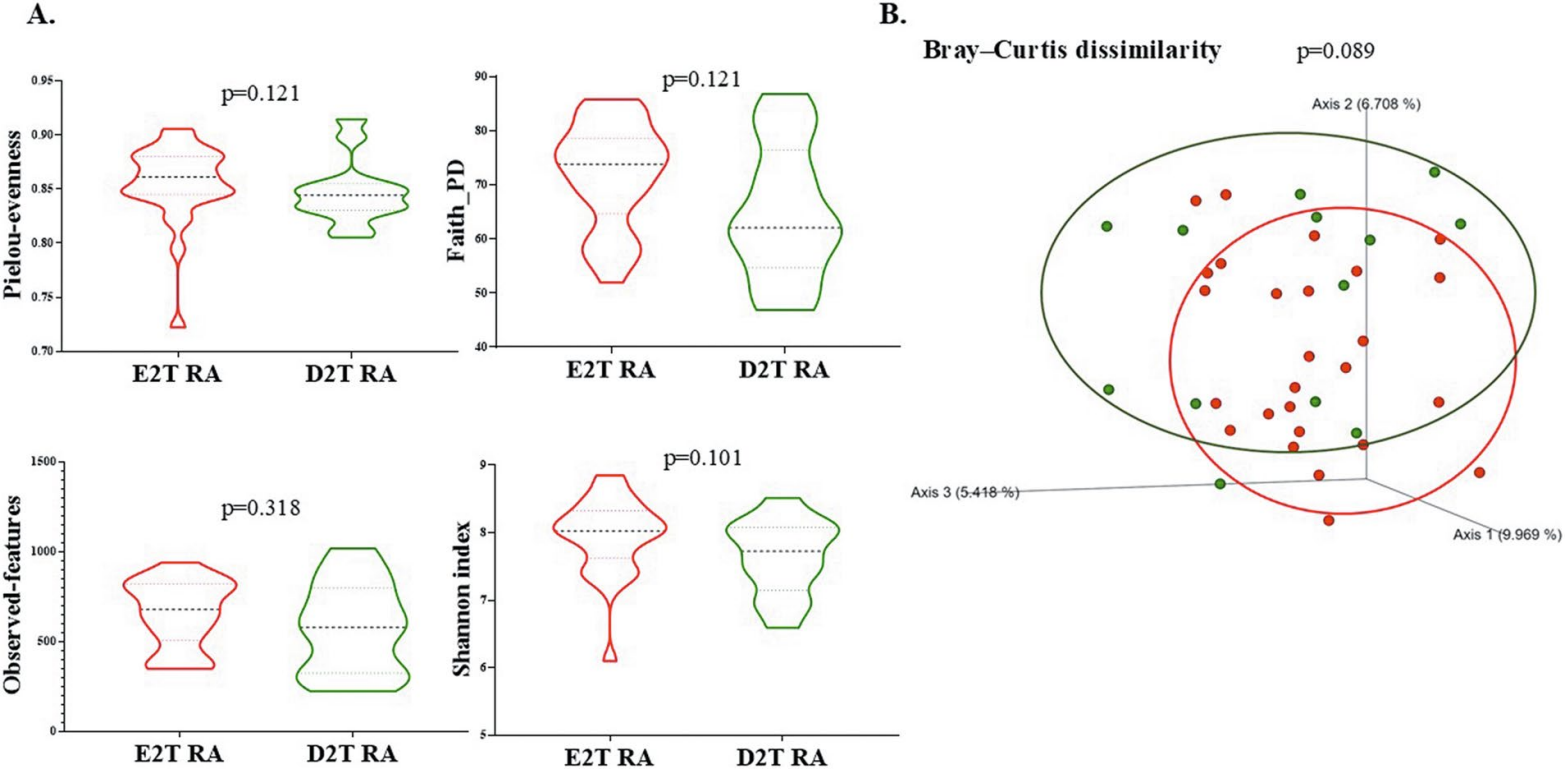
Diversity of gut microbiota between E2T RA and D2T RA patients. **(A)** Alpha diversity indexes: Pielou-evenness, Faith-PD, Observed features, and the Shannon index were compared between the 2 groups. Values are presented as mean ± SD. **(B)** Principal coordinates analysis (PCoA) corresponding to the Bray–Curtis dissimilarity index (beta diversity). The statistical analysis used permutational multivariate analysis of variance (PERMANOVA, *p* < 0.05). Green dots indicate the D2T RA patients; red dots the E2T RA patients. E2T RA, easy-to-treat rheumatoid arthritis; D2T RA, difficult-to-treat rheumatoid arthritis.

There were no differences in any of the alpha diversity measured indexes (Pielou’s evenness, *p* = 0.121; Faith’s phylogenetic diversity, *p* = 0.121; observed features, *p* = 0.318; and Shannon’s diversity, *p* = 0.101) between E2T RA and D2T RA patients (Figure 1A). While microbiota populations tended to differ based on beta diversity (Bray–Curtis dissimilarity; PERMANOVA, *p* = 0.089; Figure 1B).

**Figure 2:**
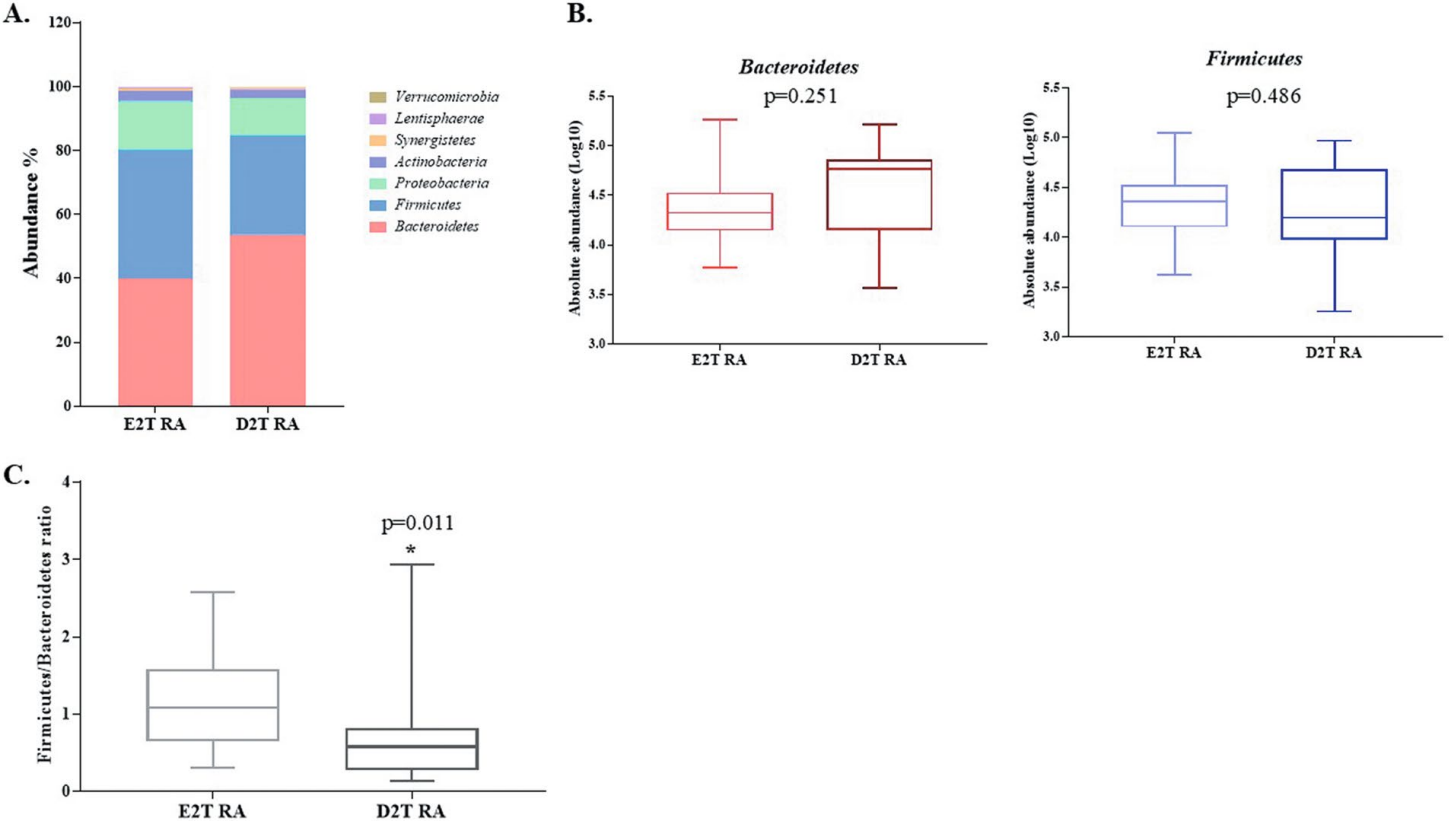
Gut microbiota analysis at the phylum level in E2T RA and D2T RA patients. **(A)** The distribution of gut microbiota at the phylum level in both RA groups. **(B)** The absolute abundance in Log10 of the phyla *Bacteroidetes* and *Firmicutes* in both RA groups. **(C)** The *Firmicutes/Bacteroidetes* ratio. * Indicates significant differences between groups (*p* < 0.05). E2T RA, easy-to-treat rheumatoid arthritis; D2T RA, difficult-to-treat rheumatoid arthritis.

At the phylum level, the dominant bacterial phyla were *Bacteroidetes*, *Firmicutes* and *Proteobacteria,* while *Actinobacteria*, *Synergistetes*, *Lentisphaerae,* and *Verrucomicrobia* accounted for smaller proportions, between 1 and 10%, in both RA groups (Figure 2A). Analysis of the abundance of *Bacteroidetes* and *Firmicutes* did not reveal significant differences between E2T RA and D2T RA (*p* = 0.251 and *p* = 0.486, respectively; Figure 2B). However, the *Firmicutes/Bacteroidetes* ratio was lower in the D2T RA patients than in the E2T RA patients (*p* = 0.011; Figure 2C).

**Figure 3:**
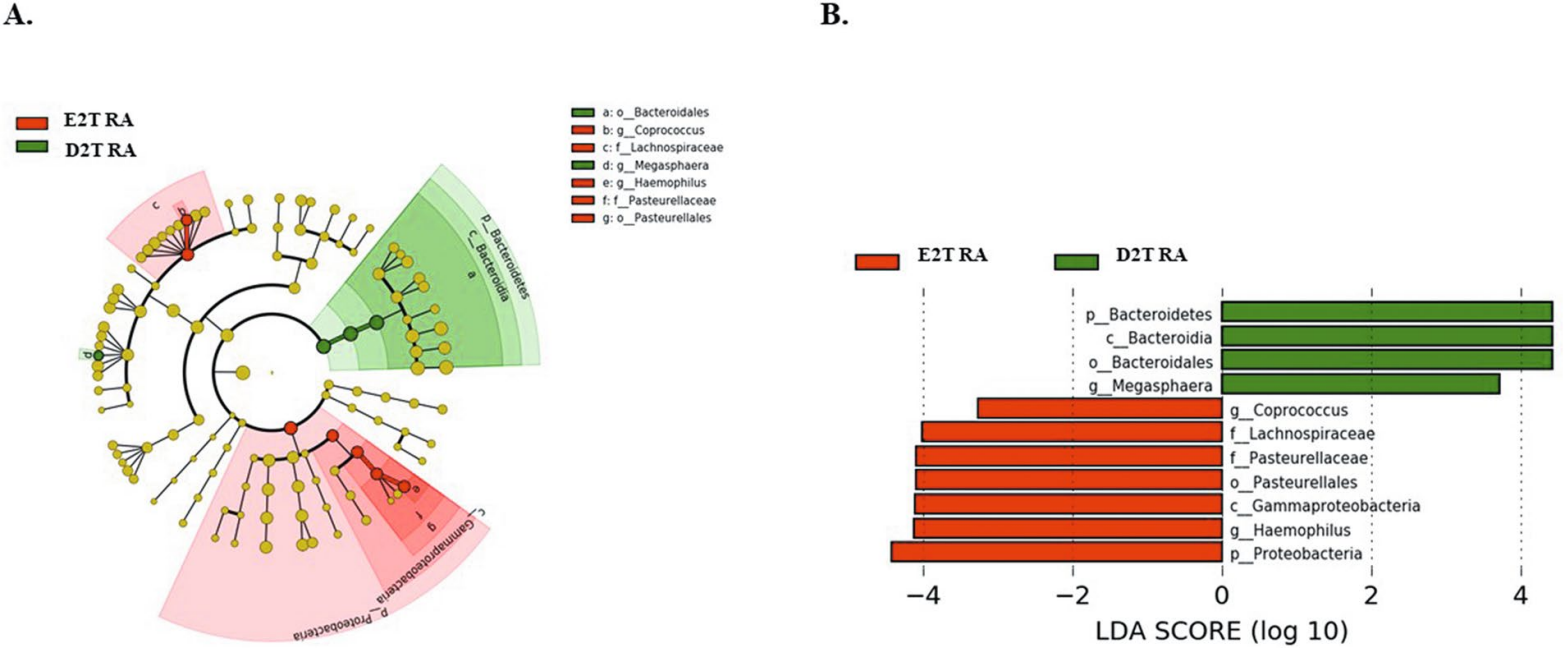
**(A)** Cladogram and **(B)** linear discriminant analysis (LDA) scores were used to determine differences in the abundance of microbes in E2T RA and D2T RA patients. LDA > 2; *p* < 0.05. E2T RA, easy-to-treat rheumatoid arthritis; D2T RA, difficult-to-treat rheumatoid arthritis.

Additionally, we performed LEfSe to identify changes in the gut microbiota between E2T RA and D2T RA patients. This analysis revealed an enrichment in the phylum *Bacteroidetes* and in the genus *Megasphaera* in the D2T RA group. In contrast, the E2T RA group exhibited a greater abundance of the phylum *Proteobacteria*, the family *Pasteurellaceae* and its genus *Haemophilus,* and the family *Lachnospiraceae* and its genus *Coprococcus* (LDA > 2; *p* < 0.05; Figure 3).

### Predicted metabolic profiles of gut microbiota

**Figure 4:**
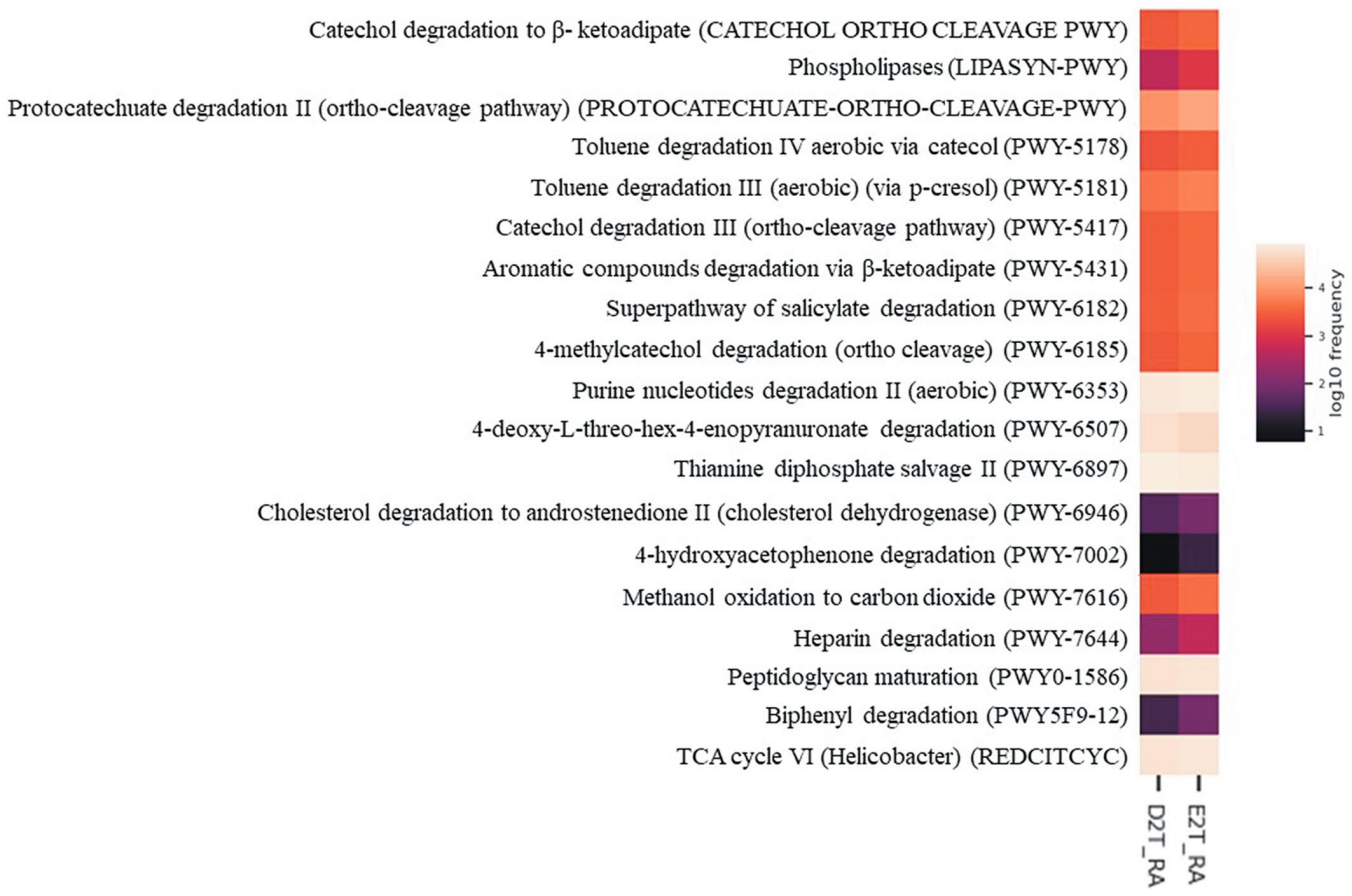
Predictive metabolic pathways by PICRUSt2. Heatmap of differentially abundant Metacyc pathways identified in the study groups (E2T RA and D2T RA patients). The values of color in the heatmap represent the normalized relative abundance of Metacyc pathways. Kruskal-Wallis test, *p* < 0.05. E2T RA, easy-to-treat rheumatoid arthritis; D2T RA, difficult-to-treat rheumatoid arthritis.

Metacyc pathway analysis was performed using PiCRUSt2 to increase our understanding of the role of gut microbiota in each of the groups studied. A Kruskal-Wallis analysis showed that 19 pathways differed between the groups (*p* < 0.05). The main pathways affected belonged to Degradation/Utilization/Assimilation pathways and Biosynthesis. In D2T RA patients, increased values were reported for the thiamine diphosphate salvage II pathway (PWY-6897) and the carbohydrate degradation pathway (PWY-6507). Increased values were also reported for E2T RA patients in the remaining pathways, some of which are involved in peptidoglycan biosynthesis and generation of precursor metabolites and energy (PWY0-1586 and REDCITCYC, respectively), and most of which are involved in degradation, specifically, aromatic compound (PWY-6185, PWY-5417, PWY-5431, PWY-7002, PWY5F9-12), toluene degradation (PWY-5178 and PWY-5181), fatty acid and lipid degradation (PWY-6946 and LIPASYN-PWY), carbohydrate degradation (PWY-7644), secondary metabolite degradation (PWY-6507), and nucleoside and nucleotide degradation (PWY-6353) (Figure 4).

### Factors associated with D2T RA patients

Table 4 shows the results of the Cox multivariate analysis (DV: D2T RA), in which 39 patients with RA were included over a mean (SD) follow-up of 103.8 (37.8) months. A total of 13/39 patients had D2T RA. The multivariate analysis showed that the *Firmicutes/Bacteroidetes* ratio was associated with a reduced risk of D2T RA (HR, 0.288; 95% CI, 0.092–0.907; *p* = 0.033), whereas the variables associated with a greater probability of D2T RA were greater average inflammatory activity according to the DAS28-ESR (HR, 2.649; 95% CI, 1.225–5.732; *p* = 0.013) and treatment with prednisone on the index date (HR, 3.794; 95% CI, 1.098–10.990; *p* = 0.008). Thus, for each 0.1-point increase in the *Firmicutes*/*Bacteroidetes* ratio, the risk of D2T RA decreased by approximately 71%.

**Table 4:** Cox regression model of factors for RA patients with refractory disease.

Dependent variable	Predictor	HR	95% CI for B	*p*-value
D2T RA*				
	Average DAS28-ESR	2.649	1.225–5.732	0.013
	Prednisone	3.794	1.098–10.990	0.008
	*Firmicutes*/*Bacteroidetes* ratio	0.288	0.092–0.907	0.033

*Refractory disease: failure of ≥ 2 lines of biologic therapy.

Variables included in the equation: sex, age, erosions, average DAS28-ESR, C-reactive protein, prednisone, body mass index, Mets, Firmicutes/Bacteroidetes ratio.

## Discussion

Patients with D2T RA continue to display symptoms after several treatment cycles, thus generating a considerable burden in clinical practice (4). Various factors are thought to affect the persistence of signs and symptoms in affected patients, which is rarely caused only by resistance to therapy (2). Today, however, there is little evidence indicating the particular characteristics, mechanisms, and factors associated with D2T RA, thus further hampering patient management (2). In an attempt to address this unmet need, the present study compares the gut microbiota profile, cumulative disease activity, and other severity-related factors between patients with D2T RA and patients with E2T RA in order to identify the intestinal microbiota profile and other factors associated with this major problem. Non-responder patients do not achieve adequate control with any treatment, while D2T RA patients have failed to respond to two or more biologics or targeted synthetic DMARDs, highlighting their clinical profile and the need for tailored therapeutic strategies.

The present study revealed that although gut microbiota populations are characterized by similar features, their abundance differed between the 2 groups. Differences were recorded in the abundance of various features, such as the families *Lachnospiraceae* and *Pasteurellaceae* and their respective genera *Coprococcus* and *Haemophilus*, which were more abundant in E2T RA patients than in D2T RA patients, in fact *Haemophilus* is implicated in RA (23). On the other hand, an increase in the abundance of *Coprococcus* has previously been observed in RA patients after therapy with methotrexate (24) and with sulfasalazine (25). Moreover, the *Lachnospiraceae* family is a major producer of butyrate (a short-chain fatty acid [SCFA]) which has beneficial effects on RA (26). In addition, a recent study (25) showed a rise in the number of SCFA-producing genera after patients with RA were treated with TNF inhibitors. These observations suggest that alterations in gut microbiota could contribute to the therapeutic effects of bDMARDs. Furthermore, this study found that the genus *Megasphaera* was more abundant in D2T RA patients. Indeed, this genus has been shown to be positively related to RA with greater abundances (27, 28).

Regarding the phyla *Firmicutes* and *Bacteroidetes*, LEfSe revealed enrichment of the phylum *Bacteroidetes* in the D2T RA group. In this line, we found that the *Firmicutes*/*Bacteroidetes* ratio was lower in D2T RA patients than in E2T RA patients. This ratio, although controversial, has been shown to be associated with different diseases; for instance, it is decreased in RA patients (29). Moreover, our multivariate analysis showed that the phylum *Bacteroidetes* was associated with a greater risk of E2T RA, whereas the phylum *Firmicutes* acted as a protective factor. The phylum *Bacteroidetes* was found to be less abundant in treated patients with established RA than in healthy controls (30, 31). In fact, as shown by Zhang et al. (32), levels of the genus *Bacteroides* were further reduced after treatment with DMARDs, especially methotrexate, which reverses the perturbations of the microbiota typically associated with RA (33, 34). In fact, several studies demonstrated that abundance of *Firmicutes* was increased while *Bacteroidetes* was decreased after treatment with methotrexate (32, 35). These changes in levels of *Bacteroidetes* could be associated with the stage of development of RA and with the response to treatment. Thus, the absence of response in patients with D2T RA could be associated with the increase we observed for the phylum *Bacteroidetes*. Moreover, in situations that alter intestinal acidity or composition, values for the phylum *Firmicutes* would decrease, thus leading to an increase in values for acetate- and propionate-producing *Bacteroidetes* (36, 37). The butyrate deficiency following the decrease in *Firmicutes* leads to a deficiency in mucin and an increase in intestinal permeability, which in turn induces a chronic inflammatory state (38). Therefore, this could be one of the factors affecting the inadequate response to treatment in patients with RA in our study.

According to the microbial metabolism approach, which was based on the inference of the metabolic pathways taken by the gut microbiota using PICRUSt2, no notable differences were found between the 2 groups studied. However, the main pathways implicated indicate that degradation is a major contributor to the results, including degradation of aromatic compounds (e.g., catechol and toluene, carbohydrates, and fatty acids), which provide energy, and that degradation was more pronounced in E2T RA patients than in D2T RA patients. Likewise, purine metabolism was increased in the E2T RA patients compared to D2T RA patients. The purine pathway plays an important role in intestinal permeability; specifically, purines help maintain a healthy energy balance and contribute to the restoration of the gut barrier (39). The findings reported may provide insight into how gut microbiota composition and metabolic activity differ between patients who respond and do not respond to treatment. This in turn could prove useful for developing targeted therapies and improving our understanding of disease mechanisms.

We also observed that patients with D2T RA had a higher average DAS28-ESR, poorer physical function according to HAQ, and higher values for inflammatory factors such as CRP and IL-6 than patients with E2T RA. Regarding these factors, the multivariate analysis showed a significant association between the average DAS28-ESR value and D2T RA. Several studies have reported that patients with D2T RA are characterized by greater inflammatory activity according to indices such as DAS28, both at initiation of biologics (40, 41) and during switches in biologic therapy over time (42). This finding could be explained by the low rates of response to treatment in patients with greater inflammatory activity (43, 44) and by the difficulty encountered when attempting to reduce very high DAS28 values until remission is achieved. Other factors that could affect higher disease activity indexes in these patients include pain and a more negative global evaluation owing to chronic disease with structural damage that has not been easily controlled. The latter observation could arise from the association between treatment with glucocorticoids and D2T RA revealed by our multivariate analysis. Reducing doses of glucocorticoids to below 5–10 mg/d has proven difficult in patients with D2T RA (4), probably because of the greater inflammatory activity. Moreover, it is worth noting that the concomitant use of glucocorticoids has been associated with severe adverse reactions and interruption of some biologics such as anti-TNF agents and anti–IL-6 agents, mainly owing to infection (45, 46).

Our study has both strengths and limitations. First, we performed a cross-sectional analysis of microbiota and inflammatory cytokines based on a single determination. However, the patients belonged to a prospective RA cohort in which all inflammation- and treatment-related data were collected longitudinally following a predetermined protocol. Second, the sample of patients with D2T RA is small, potentially limiting the possibility of detecting differences between the groups. In order to mitigate this problem, we selected 2 comparators with extreme phenotypes and twice the number of E2T RA patients per case of D2T RA. This approach enabled us to demonstrate significant differences in the main hypotheses proposed. Furthermore, while the definition of D2T RA has varied over time, we used the definition recommended by EULAR (3), which serves as a basis for most published studies. While we acknowledge that including a healthy control group would strengthen our findings, our study primarily focused on comparing D2T and E2T RA patients. Likewise, the higher proportion of female participants in our cohort may introduce confounding factors, such as hormonal influences on disease activity. Additionally, although we did not assess dietary components in detail, we observed that a high percentage of patients in both groups adhered to the Mediterranean diet. Finally, it is important to remember that other factors may affect D2T RA and have not been the object of this study, for example, metabolic differences between conventional synthetic disease-modifying antirheumatic drugs (csDMARDs) and biological or targeted synthetic disease-modifying antirheumatic drugs (b/tsDMARDs) or adherence to treatment. Nevertheless, we identified and described, for the first time, the association between microbiota-related factors and D2T RA by combining these findings with other clinical characteristics.

## Conclusion

This study found that the gut microbiota profile differs between D2T RA and E2T RA patients. Specifically, patients with D2T RA were characterized by enrichment of the phylum *Bacteroidetes* and the genus *Megasphaera*, whereas in patients with E2T RA, the phylum *Proteobacteria*, the family *Pasteurellaceae* and its genus *Haemophilus*, and the family *Lachnospiraceae* and its genus *Coprococcus* were more abundant. The *Firmicutes*/*Bacteroidetes* ratio was lower in patients with D2T RA. In addition, an increase in this ratio was seen to be an independent factor for reduced risk of D2T RA, suggesting that gut dysbiosis plays a role in nonresponse to treatment. Moreover, the above-mentioned metabolic pathway analysis revealed differences in the pathways involved in degradation of aromatic compounds, carbohydrates, and fatty acids between D2T RA and E2T RA patients. Greater inflammatory activity and use of prednisone were associated with D2T RA. The identification of new factors associated with D2T RA is a relevant finding that enhances our knowledge of patients with this disease, which is currently a severe problem with high social and health care costs. A more individualized approach including these factors can improve outcomes and reduce the risk of adverse effects of medication.

## Data Availability

The datasets presented in this article are not readily available because according to the data regulations and ethical considerations, the datasets generated and analyzed during our study cannot be made public due to the fact participants only provided their consent to the original team of investigators for the use of their data, and this information may compromise their consent to participate in the study. Requests to access the datasets should be directed to the corresponding author.

## Footnotes

^1^ http://huttenhower.sph.Harvard.edu/galaxy/
